# RandomiSed clinical trial assessing Use of an anti-inflammatoRy aGent in attenUating peri-operatiVe inflAmmatioN in non-meTastatic colon cancer – the S.U.R.G.U.V.A.N.T. trial

**DOI:** 10.1186/s12885-018-4641-x

**Published:** 2018-08-06

**Authors:** H. Paul Redmond, Peter M. Neary, Marcel Jinih, Emer O’Connell, Niamh Foley, Rolf W. Pfirrmann, Jiang H. Wang, D. Peter O’Leary

**Affiliations:** Surguvant Research Centre, Cork University Hospital, Cork, Ireland

**Keywords:** Inflammation, Colon cancer, Peri-operative, Metastasis, Recurrence

## Abstract

**Background:**

Peri-operative inflammation has been extensively highlighted in cancer patients as detrimental. Treatment strategies to improve survival for cancer patients through targeting peri-operative inflammation have yet to be devised.

**Methods:**

We conducted a multi-centre, randomised controlled clinical trial using Taurolidine in non-metastatic colon cancer patients. Patients were randomly assigned to receive Taurolidine or a placebo. The primary endpoint for the study was the mean difference in day 1 IL-6 levels. Secondary clinical endpoints included rates of post-operative infections and tumor recurrence.

**Results:**

A total of 293 patients were screened for trial inclusion. Sixty patients were randomised. Twenty-eight patients were randomised to placebo and 32 patients to Taurolidine. IL-6 levels were equivalent on day 1 post-operatively in both groups. However, IL-6 levels were significantly attenuated over the 7 day study period in the Taurolidine group compared to placebo (*p* = 0.04). In addition, IL-6 levels were significantly lower at day 7 in the Taurolidine group (p = 0.04). There were 2 recurrences in the placebo group at 2 years and 1 in the Taurolidine group. The median time to recurrence was 19 months in the Placebo group and 38 months in the Taurolidine group (*p* = 0.27). Surgical site infection was reduced in the Taurolidine treated group (*p* = 0.09).

**Conclusion:**

Peri-operative use of Taurolidine significantly attenuated circulating IL-6 levels in the initial 7 day post-operative period in a safe manner. Future studies are required to establish the impact of IL-6 attenuation on survival outcomes in colon cancer.

**Trial registration:**

The trial was registered with EudraCT (year = 2008, registration number = 005570–12) and ISRCTN (year = 2008, registration number = 77,829,558).

## Background

Peri-operative inflammation is a phenomenon that has been extensively highlighted in cancer patients as a potential therapeutic target [1–3]. Strong links have been demonstrated between the pro-inflammatory components of the peri-operative inflammatory milieu and their effects locally on residual tumor cell deposits and systemically on disseminated tumor cells [4–9]. However, treatment strategies aimed at potentially improving survival for cancer patients by targeting peri-operative inflammation have yet to be devised, with the majority of treatment strategies aimed only at the neoadjuvant and adjuvant period.

The peri-operative inflammatory response itself is vital for the healing process, however several components of the inflammatory cascade initiated by surgical trauma confer accelerant effects on residual tumor deposits [10–15]. In particular, the pro-inflammatory cytokine IL-6 has been demonstrated to act as a key mediator in tumor cell growth by upregulation of metastatic gene expression and further stimulation of down-stream pro-inflammatory cytokines and growth factor release [16]. Tumor derived IL-6 also acts as a chemoattractant to circulating tumor cells and facilitates self-seeding of disseminated tumor cells [17, 18]. Moreover, elevated levels of IL-6 carry prognostic implications in certain tumor phenotypes including colon cancer, with elevated IL-6 levels closely associated with increasing tumor size, tumor stage, presence of metastatic disease and reduced survival [19].

Up to 30% of non-metastatic colon cancer patients can develop distant metastases. In particular, 70% of metastases will occur within 2 years of the initial ‘curative’ operation. This pattern is thought to relate to the effects of surgical inflammation [20, 21]. The best illustration of this cause-effect relationship is demonstrable where complications such as anastomotic leakage occur or where conversion from laparoscopic to open surgery is necessary [22]. In these scenarios patients experience a more exaggerated inflammatory response and ultimately have a worse outcome [23, 24]. Thus, colon cancer offers an ideal model to investigate the potential therapeutic effects of targeting inflammation.

To explore the concept of attenuating inflammation safely in cancer patients undergoing major surgery we chose the ubiquitously active agent Taurolidine which has been extensively studied in a variety of clinical states involved in inflammation and cancer with a remarkable safety record [25]. Taurolidine itself possesses both anti-inflammatory and anti-neoplastic properties and has an excellent safety profile [26–28]. Our pre-clinical experience of the anti-inflammatory effects of Taurolidine were in an experimental pancreatitis model where Taurolidine reduced the endotoxin levels in an animal model [29]. Other groups have shown a reduction in pro-inflammatory mediators including IL-1 and TNF-α associated with Taurolidine administration [30]. In addition, our group has demonstrated previously in the setting of a randomised clinical trial that peri-operative IL-6 can be safely and successfully targeted using this anti-inflammatory agent in patients undergoing coronary artery bypass surgery [31].

On this basis we hypothesised that peri-adjunctive utilisation of a dual anti-inflammatory and anti-neoplastic agent, Taurolidine, could potentially reduce immediate peri-operative inflammation which may confer survival benefits for colon cancer patients. Thus, to test this hypothesis, we performed a randomised, controlled clinical trial to examine the efficacy of using an anti-neoplastic agent on peri-operative inflammation in non-metastatic colon cancer patients undergoing resection of their primary tumor. We conceptualised the term ‘surguvant’ to define therapeutic modification used in combination with surgical treatment during the peri-operative period. We also sought to examine patient safety peri-operatively and observe the effects of this treatment strategy on disease free survival.

## Methods

### Trial design

A randomised, multicentre, placebo controlled, open label clinical trial was performed. Three centres recruited patients including Cork University Hospital, Bons Secours Cork and Mercy University Hospital. Patients were randomised on a 1:1 allocation ratio to 2% Taurolidine infusions or to a placebo, given 4 times a day for a total of 4 days. A sealed envelope method was used for randomisation. Randomisation codes were generated from www.randomization.com.

The investigational medicinal product was an intravenous formulation of 2% Taurolidine (C_7_H_16_N_4_O_4_S_2_) manufactured by Geistlich-Pharma AG, CH 6110 Wolhusen/Luzern, Switzerland. The comparator placebo was 0.9% saline. The Taurolidine solution required central administration and all patients randomised to receive Taurolidine had either a central line or a peripheral long line inserted prior to the operation. First dose of Taurolidine or Placebo was administered at induction of anaesthesia. Trial bloods were performed pre-operatively, and at 3 h, 6 h, day 1, day 2, day3, day 5 and day 7 (only if still an inpatient) post-operatively. Human IFN-γ, IL-1β, IL-2, IL-6, IL-10, TNF-α, Human VEGF, Human CRP levels were measured using a customised ELISA kit manufactured by MSD (Meso Scale Discovery)® (Gaithersburg, Maryland, US).

### Inclusion/exclusion criteria

Inclusion criteria included males and females between 18 and 85 years of age with non-metastatic colon cancer. Patients undergoing elective surgery only were included.

Exclusion criteria were as follows - Rectal cancers (defined as a tumor < 15 cm from the anal verge), patients with a known allergy to taurolidine / taurine, pregnant and lactating women, evidence of underlying liver disease (abnormal LFT’s (> × 2 normal), INR > 1.5), evidence of underlying renal disease (creatinine > 180 for women, > 150 for men), blood dyscrasia (neutropenia < 1500 cells /cm3, thrombocytopenia < 100,000 cells/cm3), evidence of intestinal obstruction, metastases (M1:Distant spread or Dukes D), morbid obesity (body mass index > 40 kg/m^2^), operative risk > ASA – III, previous cancer / malignant disease other than non-melanoma skin cancer, coexisting active inflammatory disorder (including active RA, IBD, SLE), corticosteroids usage, immunosuppressive drugs, previous diagnosis of HIV, chronic active Hepatitis B or C (testing not required for study), active infection at the time of surgical intervention.

### Endpoints

The primary endpoint for the study was the difference in mean plasma IL-6 levels on day 1 in Taurolidine as compared to placebo group, adjusted for pre-operative IL-6, age and gender. Secondary laboratory endpoints included the difference in mean IL-6, IL-10 and CRP measured on post- operative days 2, 3, 5 and 7 in the Taurolidine as compared to the placebo group, adjusted for baseline measurement, age, gender and procedure type. Exploratory analyses were conducted examining levels of TNF-α, VEGF, IL-1β, IFN-γ, surface expression of CD14 and CD11b on neutrophils/monocytes, and plasma levels of C-reactive protein at the above time points.

Secondary Clinical Endpoints included a comparison of Taurolidine to control group with regard to occurrence & severity of post-operative infections, time to bowel functional recovery, post-operative pain control and recurrence (defined as local or metastatic growth) of tumor growth.

### Trial oversight

All patients provided full written informed consent. Trial participation was approved in all 3 study sites by the Cork Research Ethics Committee and the Irish Medicines Board and was registered with EudraCT (*registration number = 2008–005570-12*) and ISRCTN (*registration number = 77,829,558*). The trial was conducted in accordance with the provisions of the Declaration of Helsinki. A trial monitor was utilised to ensure the accuracy of the data collected and the case report form from each patient.

### Sample size

Precise sample size and power estimation for the trial was limited by lack of data within available literature describing expected baseline and follow-up levels of the various biochemical outcome parameters in the proposed study population and lack of available evidence regarding the efficacy of the intervention within this setting. Based on the changes in IL-6 levels following colonic resection observed by Salvadora Delgado, M.D. et al. [32], we estimated that 28 patients in each arm would allow us to detect a 0.75 SD difference in study arms with 80% power, given a type 1 error rate of 5%, using a two sample t-test with equal samples sizes and a shared variance.

### Statistical analysis

Categorical data were described by their counts and percentages in each category. Continuous variables were described by their medians and inter-quartile ranges. All continuously measured laboratory endpoints were log transformed. Differences between study arms for laboratory endpoints, at each time point specified in the protocol, were estimated using ANCOVA, with adjustment for baseline measurement, age, sex, and procedure. Differences between study arms in linear trajectories of laboratory endpoints across all time-points (days 1–7) were estimated using linear mixed effects models using a treatment arm by Time interaction term. These models were adjusted for age, sex, and procedure type. The statistical significance of the interaction was tested using the *p*-value from the likelihood ratio chi-squared test.

Differences between study arms for categorical clinical endpoints were assessed using the chi-squared test. Differences between study arms for time-to-recurrence and mortality were estimated using the Cox proportional hazards model, adjusted for age, sex, and procedure.

All analyses were conducted on an intent-to-treat basis, using the R Project for Statistical Computing (version 3.2.2 R Foundation for Statistical Computing, Vienna, Austria. www.r-project.org/).

## Results

**Fig. 1:**
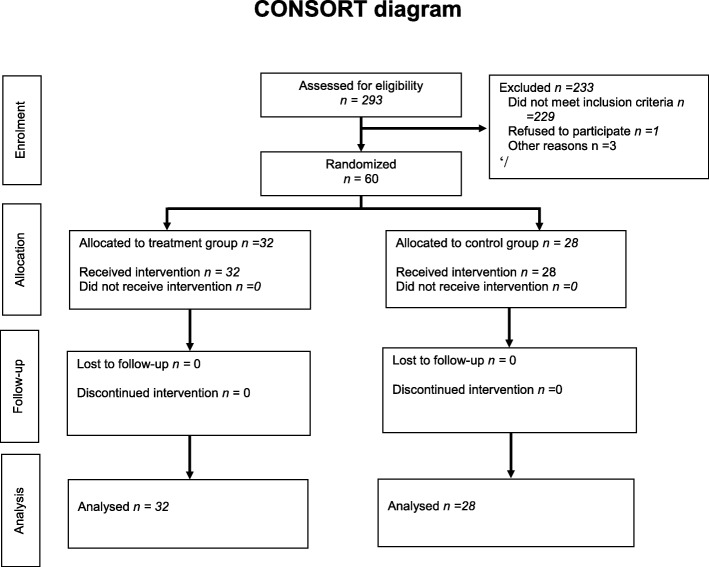
Consort diagram

A total of 293 patients were screened for trial inclusion. Two hundred thirty three patients were excluded, thus 60 patients were randomised to either study group (Fig. 1). Twenty-eight patients were randomised to placebo and 32 patients to Taurolidine. The patient, tumor and operative characteristics are summarised in Table 1. There was no significant difference in these characteristics between the two study arms.

**Table 1:** Patient, surgery and tumor characteristics, reported as n(%), or median[IQR]

	Total (*n* = 60)	Saline (*n* = 28)	Taurolidine (*n* = 32)	*p*-value
Sex				
F	21 (35%)	6 (21.4%)	15 (46.9%)	0.07
M	39 (65%)	22 (78.6%)	17 (53.1%)	
Age	69 [59.8, 72.2]	67 [58.8, 72]	69.5 [65.2, 72.2]	0.49
Surgery				
Anterior resection	27 (45%)	12 (42.8%)	15 (55.6%)	0.55
Right hemicolectomy	23 (38.3%)	12 (42.8%)	11 (40.7%)	
Total colectomy	1 (1.7%)	0(0%)	1 (3.7%)	
Other	9 (15%)	4 (14.2%)	5 (15.6%)	
Procedure				
Converted	6 (10%)	3 (10.7%)	3 (9.4%)	0.59
Lap	47 (78.3%)	23 (82.1%)	24 (75%)	
Open	7 (11.7%)	2 (7.1%)	5 (15.6%)	
Primary tumor				
T1	4 (6.7%)	1 (3.6%)	3 (9.3%)	0.59
T2	8 (13.3%)	5 (17.8%)	3 (9.3%)	
T3	34 (56.6%)	16 (57.1%)	18 (56.2%)	
T4	8 (13.3%)	2 (7.1%)	6 (18.8%)	
T4a	4 (6.7%)	3 (10.7%)	1 (3.2%)	
T4b	1 (1.7%)	1 (3.5%)	0 (0%)	
Carcinoid	1 (1.7%)	0 (0%)	1 (3.2%)	
Regional lymph nodes				
N0	31 (51.6%)	12 (42.8%)	19 (59.4%)	0.37
N1	9 (15%)	5 (17.8%)	4 (12.5%)	
N1a	7 (11.6%)	4 (14.2%)	3 (9.4%)	
N1b	6 (10.0%)	3 (10.8%)	3 (9.4%)	
N2	5 (8.4%)	3 (10.8%)	2 (6.2%)	
N2b	2 (3.4%)	1 (3.6%)	1 (3.1%)	
Lymph node yield	17	16	18	0.58
Follow-up (months)	34	32	37	0.23

### Primary endpoint and post-hoc analysis

**Fig. 2:**
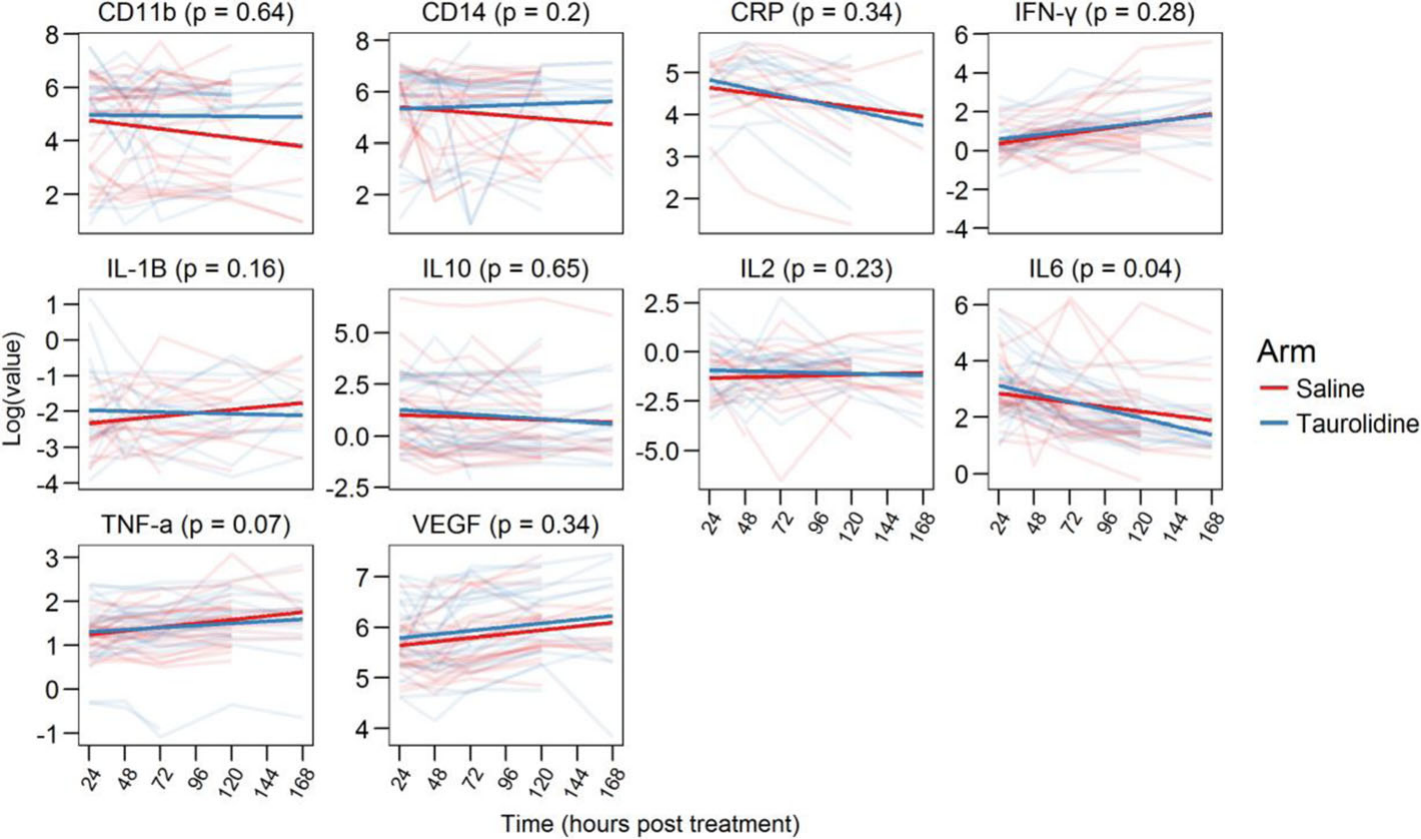
Seven-day linear trends in laboratory endpoints. Differences in the 7-day linear trend between treatment arms were tested using linear mixed effects models and treatment X time interaction term. The *p*-values in the plot are from the likelihood ratio test for a model including that interaction term vs. a model without it

A peak in IL-6 levels in both study groups was evident at 24 h post surgery. The overall trend for this IL-6 peak was to settle over the remaining study days. IL-6 levels at 24 h were equivalent in the Taurolidine and placebo group (*p* = 0.89). Post-hoc analysis was performed to analyse the selected study cytokines in both study groups over the entire 7 day study period (Fig. 2). IL-6 levels were found to be significantly attenuated in the Taurolidine group compared to the placebo group over the course of the study period (*p* = 0.04). In addition, the mean levels of IL-6 were significantly attenuated in the Taurolidine group compared to placebo at 7 days (*p* = 0.04).

### Secondary laboratory end-points

TNF-α levels demonstrated a peak at 24 h post surgery. The rate of TNF-α attenuation in Taurolidine treated patients compared to placebo over the 7 days approached significance (*p* = 0.07). At the 7 day time point, IL-2 demonstrated a trend towards attenuation in the Taurolidine treated group compared to placebo (*p* = 0.06). The remaining cytokines and growth factors did not show any significant changes over the study period between the two study groups.

### Secondary clinical end-points

The median length of stay was 6 days for the entire study cohort. There were 11 cases of post-operative infective complications. Six of the infective complications were surgical site infections, 5 cases were in the placebo group and 1 in the Taurolidine group (*p* = 0.09) (Table 2). Three post-operative infections were anastomotic leaks, 2 in the placebo and 1 in the Taurolidine treated group (*p* = 0.41). The remaining 2 infective complications consisted of a lower respiratory tract infection in a patient with underlying COPD and cellulitis at the site of PICC line insertion. Both of these infective complications were in the Taurolidine group.

**Table 2:** A comparison of clinical end-point data

		Placebo (*n* = 28)	Taurolidine (*n* = 32)	*p*-value
Pain	Day 1	2.5	1.8	0.391
	Day 2	1.9	2	0.873
	Day 3	1.9	1	0.179
Time to bowel function (hrs)		39	34	0.32
Infective complication		8	4	0.19
Surgical site infection		5	1	0.09
Anastomotic leak		2	1	0.59
Other		0	2	0.49

The mean time to return of bowel function was 39 h in the placebo group and 34 h in the Taurolidine group (*p* = 0.32). Pain scores demonstrated no difference between the two study groups (*p* = 0.39).

### Survival end-points

At the 2 year follow-up time point there were 3 recurrences in total, 2 in the placebo group and 1 in the Taurolidine group (*p* = 0.38) (Table 3). The overall median follow-up at the time of data analysis was 34 months (range 24 months to 5 years). The median follow-up time in the placebo group was 32 months (range 24–76 months). The median follow-up time in the Taurolidine group was 37 months (range 24–76 months). In this time period 6 patients had experienced a recurrence, with 3 in the placebo treated group and 3 in the Taurolidine group (*p* = 0.64). In these patients the median time to recurrence was 19 months in the placebo group and 38 months in the Taurolidine group (*p* = 0.27). The 3 placebo group patients developed loco-regional recurrence. The 3 Taurolidine patients developed distant metastatic disease.

**Table 3:** A comparison of survival outcome data

	Total (*n* = 60)	Saline (*n* = 28)	Taurolidine (*n* = 32)	*p*-value
Overall recurrence	6	3	3	1
Recurrence at 2 years	3	2	1	0.389
Mean time to recurrence (months)		16.3	28.6	0.4
Median time to recurrence (months)		19	38	0.268

## Discussion

This multi-centre, randomised clinical trial was specifically designed to address the question of the potential effects of surgically induced inflammation on perioperative tumor kinetics using IL-6 as a surrogate marker. We used the agent Taurolidine to therapeutically modify these effects during the peri-operative time period. Although there was no difference seen at the early 24 h time-point, post hoc analysis demonstrated a significant trend for IL-6 attenuation over the 7 day post-operative period in Taurolidine versus the control group, with levels being significantly different at post-operative day 7. Thus whilst we do not demonstrate an immediate post-operative effect, in post hoc analysis we see a delayed effect from Taurolidine on circulating Il-6 levels.

The levels of circulating colon cancer cells and circulating colon cancer stem cells are significantly higher peri-operatively, particularly within the portal venous system [33, 34]. Together with a surge in circulating pro-inflammatory cytokine and growth factor levels, this is a significant and completely understudied phenomenon that potentially has detrimental implications for cancer patients in both the short and long term. This trial provides evidence, at least in part, that targeting the inflammatory response in particular can potentially reduce post-operative tumor metastatic growth resulting in improved patient survival outcome. In particular it can reduce inflammation and ultimately may improve patient outcome.

Circulating levels of IL-6 were attenuated in response to Taurolidine administration in a time dependent manner. It is possible that earlier administration of Taurolidine a number of days pre-operatively might result in a more immediate attenuation of IL-6 post-operatively. Furthermore, post-operative administration over an extended period of time may also have an added benefit in the presence or absence of chemotherapy [35].

IL-6 can propagate colon cancer cell growth and unsurprisingly circulating levels of IL-6 are prognostic in colon cancer [36]. The present study was not powered for a formal survival analysis so it is not possible to assess the clinical impact of IL-6 attenuation, however interesting trends are emerging from this data in relation to survival outcome. The median time to tumor recurrence was longer, though not significantly different in patients who were treated with Taurolidine versus those that were treated with placebo (38 months versus 19 months). However, these are only trends and would require an appropriately powered trial in order to draw solid conclusions.

Surgical patients are dependent upon components of inflammation to heal safely. Compromise of the healing process can lead to life-threatening complications, for example an anastomotic leak. Unsuccessful attempts at utilising the peri-operative period for adjunctive therapies have failed in the past due to compromise of safety. The present trial demonstrates that the anti-inflammatory agent Taurolidine can target key pro-inflammatory cytokines without compromising patient safety.

Several key points now need to be addressed. Firstly, does extension of the period of Taurolidine administration help to further attenuate pro-inflammatory responses to surgical trauma. Careful attention to patient safety and safety outcomes will be required if further extension of the administration period is considered. Secondly, does the attenuation of the inflammatory response translate into a survival benefit? We hypothesise that the attenuation of Il-6 levels over the initial post-operative week may lead to a clinically relevant improvement in patient outcomes as the peri-operative interaction of disseminated tumor cells and the pro-inflammatory milieu of cytokines/growth factors will occur in a less favourable environment. However this needs to be further addressed in a large, adequately powered clinical trial.

## Conclusions

Peri-operative use of Taurolidine attenuated circulating IL-6 levels in a progressive manner post-operatively. We believe that further investigation of such ‘surguvant’ therapies during this under-investigated period could lead to significant improvements in surgical patient outcomes in a safe manner.

## Abbreviations

ANCOVA: Analysis of covariance
ASA: American society of anaesthesiologists
CD-11b: Cluster of differentiation 11b
CD-14: Cluster of differentiation 14
COPD: Chronic obstructive pulmonary disease
CRP: C-reactive protein
HIV: Human immunodeficiency virus
IBD: Inflammatory bowel disease
IFN-γ: Interferon gamma
IL-10: Interleukin 10
IL-1β: Interleukin 1-beta
IL-2: Interleukin 2
IL-6: Interleukin-6
INR: International normalised ratio
LFTs: Liver function tests
RA: Rheumatoid arthritis
SLE: Systemic lupus erythematous
TNF-α: Tumour necrosis factor-α
VEGF: Vascular epithelial growth factor
